# Sustainable health—a call to action

**DOI:** 10.1186/s44263-023-00007-4

**Published:** 2023-08-03

**Authors:** Rhoda K. Wanyenze, Tobias Alfvén, Rawlance Ndejjo, Nina Viberg, Karin Båge, Charles Batte, Daniel Helldén, Helena Lindgren, Roy William Mayega, Grace Ndeezi, Stefan Swartling Peterson, Barnabas Nawangwe, Ole Petter Ottersen

**Affiliations:** 1 Department of Disease Control and Environmental Health, School of Public Health, College of Health Sciences, Makerere University, P.O Box 7072, Kampala, Uganda; 2 Centre of Excellence for Sustainable Health, a Collaboration Between Makerere University and Karolinska Institutet, Kampala, Uganda; 3 Department of Global Public Health, Karolinska Institutet, 171 77 Stockholm, Sweden; 4 Centre of Excellence for Sustainable Health, a Collaboration Between Makerere University and Karolinska Institutet, Stockholm, Sweden; 5 Lung Institute, College of Health Sciences, Makerere University, P.O Box 7072, Kampala, Uganda; 6 Department of Women’s and Children’s Health, Karolinska Institutet, 171 77 Stockholm, Sweden; 7 Department of Epidemiology and Biostatistics, School of Public Health, College of Health Sciences, Makerere University, P.O Box 7072, Kampala, Uganda; 8 Department of Paediatrics and Child Health, School of Medicine, College of Health Sciences, Makerere University, P.O Box 7072, Kampala, Uganda; 9 Makerere University, P.O Box 7062, Kampala, Uganda; 10 Karolinska Institutet, 171 77 Stockholm, Sweden

## Abstract

*Sustainable* *health*, a multisectoral area for study, research, and practice towards improving health and well-being for all while staying within planetary boundaries, is a prerequisite to reaching the 2030 agenda and the work and journey towards a world in which everyone, everywhere can live a healthy and fulfilled life.

## Background

Progress in achieving the United Nations’ 2030 Agenda for Sustainable Development has been alarmingly slow. The action gap is glaring, and we urgently need a new approach to reaching the highly interlinked Sustainable Development Goals (SDGs) and health for all. The COVID-19 pandemic cast a stark spotlight on pre-existing local and global inequities and inequalities that, while acknowledged, have not been sufficiently prioritised in our endeavour to realise the SDG ambitions [1]. Societies demonstrated remarkable resolve in overcoming the pandemic, an approach that should be applied to other challenges to health and society. The Intergovernmental Panel on Climate Change 2022 report underscored the increase in climate change catastrophic events and their effects on health [2]. Other global trends such as antimicrobial resistance, zoonotic diseases, violence, and pandemics affect the health and well-being of present and future generations and sustainable development [3]. Interrupting these negative trends demands immediate and sustainable solutions with global transformations beyond the capacity of any single stakeholder or country. Humanity must decisively act together through equitable and sustainable partnerships to rethink and accelerate actions towards the 2030 Agenda and the broader goals of health equity, while staying within the “planetary boundaries”, i.e. the environmental limits within which humanity can safely operate [4]. We must aim for a world in which everyone, everywhere, can live a healthy, fulfilled life in a socially, economically, and ecologically sustainable way. Building on the definitions of Global Health [5] and sustainable development [6], we define s*ustainable health* as a multisectoral area for study, research, and practice towards improving health and well-being for all, while staying within planetary boundaries. In this article, we explore the key transformations necessary and present a call to action for various stakeholders.

## Rethinking the approach to achieving health and well-being

The 2030 Agenda is holistic with profound and complex interactions across the SDG domains. Health and well-being (SDG 3) is linked to every sector of society and ultimately to the health of the planet. Without achieving SDG 3 in a sustainable way, we will not achieve sustainable societies and vice versa. *Sustainable health* means acknowledging the importance of these interconnections and how the health gains today will affect future generations. As global trends often disproportionately impact marginalised groups and individuals, we must focus on those who are most at risk of being left behind to eliminate inequities that affect health outcomes. Policies and interventions developed and implemented from this perspective will ensure equitable and *sustainable health* for both people and the planet and significantly impact other development goals in the 2030 Agenda.

## Rethinking the relationship with the planet

Health and well-being of current and future generations and that of the planet are inextricably linked [7]. By unsustainably exploiting natural resources, human civilisation has flourished in some societies while in others it has led to the degradation of nature’s life support systems and to significant health challenges whose consequences will continue. This poses challenges to global health gains achieved in recent decades and is likely to become increasingly worrisome during the second half of this century and beyond [8]. The WHO estimates that the climate crisis already causes approximately 250,000 additional deaths per year due to, for example, heat stress, malaria, diarrhoea, and malnutrition. Health holds a central place in the vision for a better world where societies ensure *sustainable health* and equitable access to health systems that meet their health needs. However, this must be achieved without compromising the ability of future generations to meet their own needs [6]. Respecting planetary boundaries is key to achieving equitable *sustainable health* for current and future generations.

## Rethinking partnerships

Fundamental changes in the current working methods must be made to progress towards *sustainable health*. The multilateral organisations that form part of governance structures for health today are fragmented, often sector-specific, and as such inadequate and under-resourced to handle the cross-sectoral and interconnected challenges impeding the achievement of *sustainable health* and the 2030 Agenda [9]. The intellectual, financial, and political centres of Global Health also remain firmly established in high-income countries, for example, of the individuals seated on boards across 146 leading global health organisations in 2022, only 2.5% were nationals of low-income countries [10]. Researchers from low- and middle-income countries (LMICs) tend to be disadvantaged when it comes to science and authorship of global health articles. Moreover, “trickle-down science”, directed by high-income countries and conducted in low-income countries is still a reality indicating imbalances in power relations in global health. Research in line with *sustainable health* implies shifting from “research on” to “research with and for”. The 2030 agenda acknowledges that all countries have a unique set of challenges and threats to manage and solve in order to achieve sustainable health and development. Any country can be hit by unexpected health threats, which calls for resilient health systems and societies, equitable partnerships, and use of bottom-up approaches and reciprocal learning—a recognition of global interconnectedness and the existence of a wealth of experiences and solutions across communities. LMICs must provide leadership to define agendas and approaches and progressively diminish reliance on external systems. Reciprocal and equitable relationships and collaborations are the foundations of *sustainable health*. Sustainable health also requires a global governance for health that is transformed in line with the 2030 agenda.

## Call to action

**Fig. 1:**
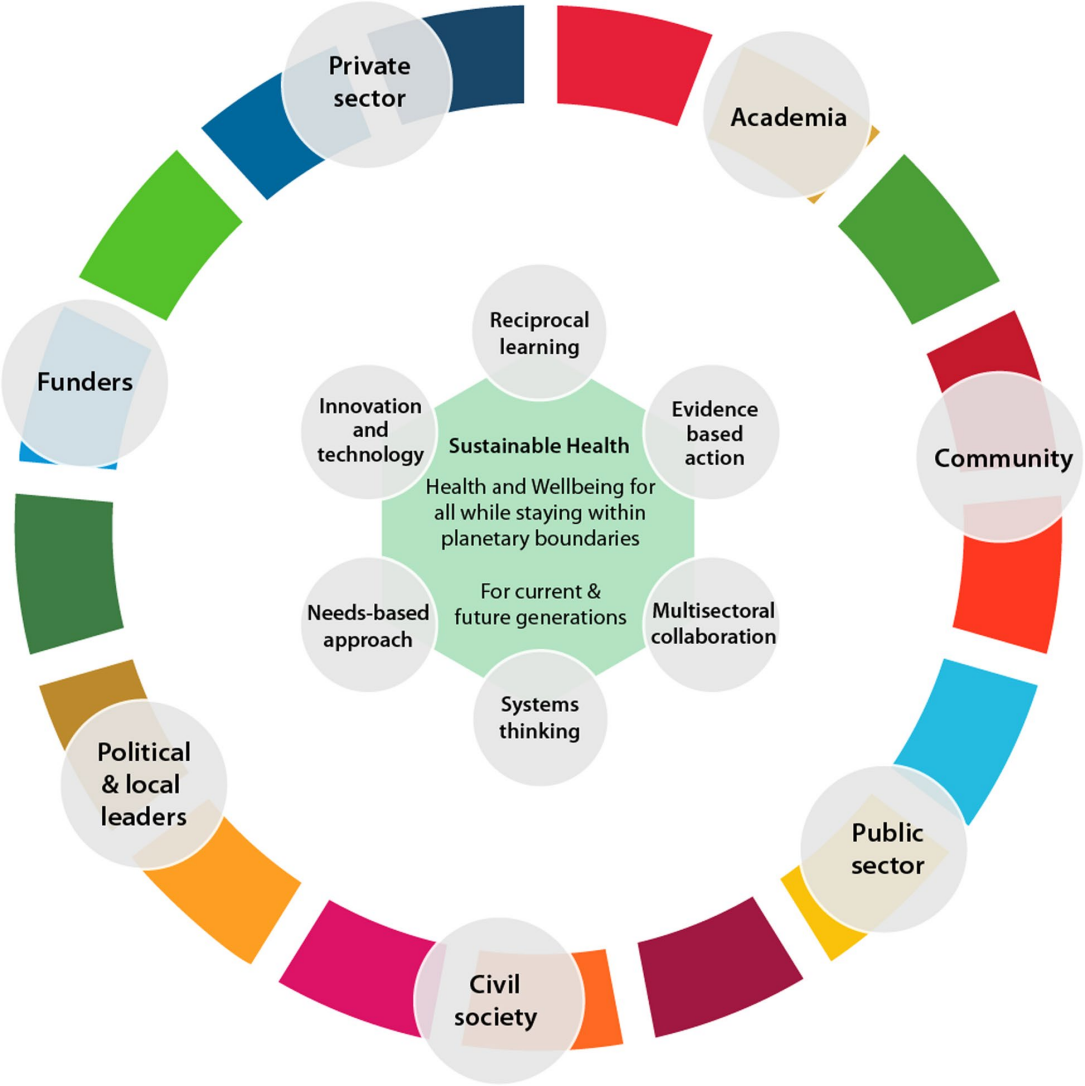
Actions of several stakeholders are required for sustainable health

Many of the challenges described might seem obvious. However, despite being informed, actors in the field seldom go beyond words. Achieving *sustainable health* requires coordinated actions from various stakeholders including academia, public and private sectors, civil society, political and local leaders, funders, and the community (Fig. 1). For academia, institutions should integrate sustainable health concepts in training, enabling students to develop multidisciplinary and transversal competencies, as well as restructure education to involve working with communities to solve their challenges. Academic and research institutions should expand research to address evidence gaps, bridge policy-to-practice gaps, and create sustainability-friendly innovations and technologies. Moreover, institutions should train cadres of researchers that are responsive to the complexities of the health and environment nexus to create a critical mass to proactively advance the sustainable health research, policy, and service agenda. The public sector should make use of the knowledge created by academia and integrate *sustainable health* in its policies and programmes, to break silos across sectors and departments and provide funding in a way that encourages cross-sectoral collaboration. The public sector should also create relevant policies, coordination mechanisms, and data systems for tracking progress towards *sustainable health*. The private sector should adopt sustainable practices in their operations and invest in generating sustainability-focused solutions. The sector is also well-placed to create businesses that directly address environmental and social challenges. Civil society should lead the way in advocacy for *sustainable health* including lobbying for policy changes, creating community awareness, and demanding accountability from other sectors. Political and local leaders should lead priority setting for contextually relevant evidence and solutions, local ownership, and investments in *sustainable health*. Funders should consider power dynamics and strive for local relevance and predictability in funding. They should establish funding focused on exploring *sustainable health* linkages and restructuring health systems. In evaluating projects for funding, funders should require adherence to sustainability principles and consider the impact of the proposed work on the environment. The communities should take an active role in demanding commitment and accountability from all stakeholders. All sectors must lead by example by integrating sustainability into their work and instituting sustainable policies, processes, systems, and infrastructure as well as providing necessary data. Actors should collaborate with other sectors locally and globally, be open to learning, and work towards ensuring that no one is left behind.

## Leading the way

The Centre of Excellence for Sustainable Health (CESH), a partnership between Makerere University, Uganda, and Karolinska Institutet, Sweden, has been implementing activities to foster sustainable health. The centre has developed important tools dubbed Tools for Action (https://cesh.health/tools-for-action/) to provide stakeholders with guidance on how to bridge identified gaps to further progress towards *sustainable health*. The developed Tools for Action are as follows: translation of research into action, multisectoral work, information and digitalisation, visualising and communicating data, and innovation and technology for health. Through a capacity-strengthening project (https://cesh.health/projects/draft-building-capacity-for-sustainable-development-in-fragile-states-sdgcap/), the centre has been increasing awareness of *sustainable health* in academia, civil society, and public and private sectors and working with them to take steps to integrate sustainability into their work in the Democratic Republic of Congo, Somalia, and Uganda. There are other notable efforts by different stakeholders. For example, some funders now require the integration of sustainability into projects and some private sector entities are adopting recommended environmental, social, and governance principles. With all stakeholders taking a positive step, collectively, the potential for impact becomes immense.

## Conclusions

We all have a responsibility to collaborate towards *sustainable health*. We must comprehensively approach the 2030 Agenda, acknowledging that SDGs are interlinked and that our health is inseparable from the health of the planet. We must form equitable partnerships and intentionally engage in reciprocal learning across contexts and sectors. Health gains of today should and can be achieved in a way that will not negatively impact the planet nor the health of current and future generations.

## Data Availability

Not applicable.

## References

[1] Ottersen OP, Engebretsen E. COVID-19 puts the Sustainable Development Goals center stage. Nat Med. 2020;26:1672–3. 33037423 10.1038/s41591-020-1094-y

[2] Pörtner HO, et al. Climate change 2022: impacts, adaptation and vulnerability. 2022.

[3] Masson-Delmotte V, et al. IPCC, 2021: climate change 2021: the physical science basis. Contribution of Working Group I to the Sixth Assessment Report of the Intergovernmental Panel on Climate Change. 2021.

[4] Steffen W, et al. Planetary boundaries: guiding human development on a changing planet. Science. 2015;347:1259855. 25592418 10.1126/science.1259855

[5] Koplan JP, et al. Towards a common definition of global health. Lancet. 2009;373:1993–5. 19493564 10.1016/S0140-6736(09)60332-9 PMC9905260

[6] Brundtland G, Khalid M, Agnelli S, Al-Athel S, Chidzero B. Our common future. Oxford: World Commission on Environment and Development; 1987.

[7] Rockstrom J, et al. A safe operating space for humanity. Nature. 2009;461:472–5. 19779433 10.1038/461472a

[8] Whitmee S, et al. Safeguarding human health in the Anthropocene epoch: report of the Rockefeller Foundation-Lancet Commission on planetary health. Lancet. 2015;386:1973–2028. 26188744 10.1016/S0140-6736(15)60901-1

[9] Kumanan R. Global health and its discontents. Lancet. 2021;397:1543–4. 33894830 10.1016/S0140-6736(21)00713-3 PMC9754103

[10] Global Health 50/50. ‘Boards for all? A review of power, policy and people on the boards of organisations active in global health. Cambridge; 2022. https://globalhealth5050.org/2022-report/?nav&section=overview.

